# Transmission Dynamics of *Schistosoma japonicum* in the Lakes and Marshlands of China

**DOI:** 10.1371/journal.pone.0004058

**Published:** 2008-12-30

**Authors:** Darren J. Gray, Gail M. Williams, Yuesheng Li, Donald P. McManus

**Affiliations:** 1 Molecular Parasitology Laboratory, Queensland Institute of Medical Research, Herston, Brisbane, Queensland, Australia; 2 School of Population Health, The University of Queensland, Herston, Brisbane, Queensland, Australia; 3 Hunan Institute of Parasitic Diseases, WHO Collaborating Centre for Research and Control on Schistosomiasis in Lake Region, Yueyang, People's Republic of China; London School of Hygiene & Tropical Medicine, United Kingdom

## Abstract

**Background:**

*Schistosoma japonicum* is a major public health concern in China, with over one million people infected and another 40 million living in areas at risk of infection. Unlike the disease caused by *S. mansoni* and *S. haematobium*, schistosomiasis japonica is a zoonosis, involving a number of different mammalian species as reservoir hosts. As a result of a number of published reports from China, it has long been considered that bovines, particularly water buffaloes, play a major role in human *S. japonicum* transmission there, and a drug-based intervention study (1998–2003) around the Poyang Lake in Jiangxi Province provided proof of concept that water buffaloes are, indeed, major reservoirs of human infection in this setting.

**Methods and Findings:**

In this study we incorporated recently obtained epidemiological information to model the steady-state *S. japonicum* transmission as well as the impact of the removal of *S. japonicum* transmission attributable to water buffaloes on human infection rates across six different endemic scenarios within three villages in the Dongting (Hunan) and Poyang (Jiangxi) lakes of southern China. Similar results were obtained for all scenarios. Steady-state *S. japonicum* infection rates remained constant and human prevalence and incidence were predicted to fall considerably over time. The model showed that the contribution of *S. japonicum* water buffalo transmission to human infection ranged from 39.1% to 99.1% and predicted that the removal of water buffalo transmission would reduce parasite reproductive rates below 1. This indicates that without the contribution of water buffaloes, *S. japonicum* transmission is interrupted and unsustainable. These scenarios are generalizable to other endemic villages in the lake and marshland areas of China where a similar cycle of snail infection and infection/reinfection of humans and bovines occurs.

**Conclusions:**

Along with previous epidemiological data, our findings strongly support water buffaloes as an important component of the transmission cycle that affects humans in the lake and marshlands region of China, a feature which appears to differ from the situation prevalent in the Philippines where their contribution is less pronounced. Our conclusions underscore the rationale for removal, replacement or treatment of water buffaloes, and for the development and deployment of a transmission blocking buffalo vaccine against *S. japonicum* for this setting to achieve the goal of transmission control. The Chinese Government has recently commenced a new integrated national strategy to improve on existing approaches to control schistosomiasis in the lake and marshlands region by reducing bovines and humans as a source of *S. japonicum* infection to *Oncomelania* snails.

## Introduction

According to the World Health Organization, the disease burden attributable to schistosomiasis is 1.93 million Disability-Adjusted Life-Years [1], although reassessment of schistosomiasis-related disability [2], combined with recent information on the global prevalence of schistosome infection [3] suggests that the true burden of schistosomiasis is substantially greater than previously appreciated [4]. The global prevalence of schistosomiasis is currently estimated to be 207 million with another 779 million people at risk of infection [5].

*Schistosoma japonicum* is the causative agent of schistosomiasis in China, The Philippines and in small pockets of Indonesia [6], [7]. The pathology caused by *S. japonicum* infection is associated mainly with an immune response to eggs that are trapped in the tissues during the periintestinal migration or after embolisation in the liver, spleen, lungs, or cerebrospinal system. The clinical features of schistosomiasis japonica can be severe, ranging from fever, headache and lethargy, to serious fibro-obstructive pathology leading to portal hypertension, ascites and hepatosplenomegaly, which can cause premature death [6].

There are reports of cases of human schistosomiasis in China as far back as 400BC and in the 1950's schistosomiasis japonica killed and disabled millions of Chinese [6]–[8]. Despite over 50 years of intensive control efforts that included the World Bank Schistosomiasis Control Project (WBSCP) from 1992–1999, schistosomiasis still remains a major public health concern there with over one million Chinese currently infected and another 40 million living in areas at risk of infection [7]–[10]. The majority (>80%) of schistosomiasis cases occur around the Dongting and Poyang lakes and the marshland regions of Hunan, Jiangxi, Anhui, Hubei and Jiangsu in southern China; transmission occurs also in the mountainous areas of Sichuan and Yunnan [7], [10]–[14].

Unlike schistosomiasis caused by *S. mansoni* and *S. haematobium*, schistosomiasis japonica is a zoonosis. It is estimated that over 40 species of wild and domestic animals comprising 28 genera and 7 orders can be infected [15]. The range of mammalian hosts complicates schistosomiasis control efforts and, as well as the public health considerations, the disease adds to the economic burden of communities as schistosome infection debilitates domestic livestock that are used for food and as work animals [7]. It was initially believed that those of public health importance were rats, dogs, pigs, sheep and goats, cattle and water buffaloes [15], [16]. In China, however, dogs, pigs, rats and goats are likely to contribute only minimally to overall transmission. This is because dogs, until recently, were relatively uncommon in rural China [7]; pigs are short-lived, and are confined to pens within the communities so have limited water contact [7]; rats (field rats, *Rattus norvegicus*; albino rats, *R. norvegicus albus*) produce limited amounts of faeces and harbour female *S. japonicum* worms with few viable eggs [15]–[20]; and sheep and goats also have low faecal output and are only present on the marshlands for limited periods as they are sold at an early age as a food source [15], [17].

There is a substantial literature recognizing that bovines, particularly water buffaloes (*Bubalus bubalis*), play a major role in the transmission of *S. japonicum* to humans in China [7], [8], [10], [15]–[27]. Significantly, the daily faecal output from a water buffalo (∼25 kg) has been estimated to be at least 100 times that produced by a human individual (250 g) [7], [17]. Accordingly, a recent study has shown that the environmental contamination attributable to 238 infected bovines (225/13; buffaloes/cattle) was, in total, approximately 28.7 million eggs/day [24], emphasizing their considerable contribution to the deposition of *S. japonicum* eggs into the external environment. Furthermore, a praziquantel-based intervention study (1998–2003) [21] around the Poyang Lake in Jiangxi Province provided experimental proof that water buffaloes are major reservoir hosts for human *S. japonicum* infection. The trial showed that water buffalo chemotherapy impacted upon human infection rates by a greater reduction in human incidence in an intervention village (where all humans and water buffaloes were subjected to praziquantel treatment) compared with control village (human praziquantel treatment only). Mathematical modelling [28] supported this conclusion and predicted that water buffaloes were responsible for approximately 75% of human transmission in this setting [21]. Furthermore, a molecular field survey of *S. japonicum* in China using microsatellite markers showed that humans and bovines contribute considerably more to the parasite reservoir within snails than other definitive host species [29].

Here, we have modelled further the transmission dynamics of *S. japonicum* in the lake and marshland regions of southern China, using newly obtained epidemiological parameters resulting from an extensive field study in Jiangxi and Hunan Provinces [24]. Our findings reinforce the argument that water buffaloes are major reservoirs for human *S. japonicum* infection in these areas.

## Methods

**Figure 1:**
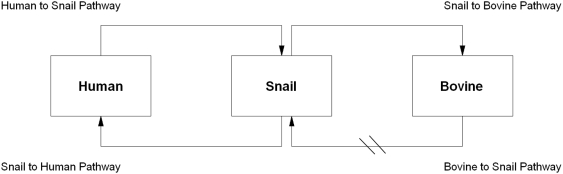
Transmission pathways of *S. japonicum* in China. The oblique lines show the blocking of the bovine to snail pathway employed in the model; this prevents miracidia that hatch from eggs excreted by bovines from infecting oncomelanid snail intermediate hosts.

Using the mathematical model developed by Williams *et al* [28] we modelled steady-state (continued transmission without intervention) *S. japonicum* transmission as well as the removal of *S. japonicum* transmission due to water buffaloes across six different endemic scenarios, so as to assess the impact of water buffaloes on human *S. japonicum* transmission. We estimated relevant model parameters from data recently collected from Hunan and Jiangxi Provinces [24], so that the predictions would apply to the transmission dynamics of the lake and marshland areas. Removal of *S. japonicum* transmission, through the blocking of the bovine to snail pathway in the *S. japonicum* transmission cycle (Figure 1), was simulated by setting the parameters governing the bovine to snail transmission to zero. Human infection rates for the simulated blockage of the bovine to snail pathway in the transmission cycle were compared with the simulated steady-state transmission.

The contribution of water buffaloes to human transmission (B_Tx_) was quantified using the formula:
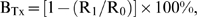
where R_0_ = Reproductive Rate (before removal of water buffalo transmission) and R_1_ = Reproductive Rate (after removal of water buffalo transmission).

### Mathematical Model

The model of Williams *et al* [28] (implemented within MATHCAD [30]), the first mathematical model published on the transmission dynamics of *S. japonicum*, was developed to simulate the transmission of schistosomiasis in China, and to predict the effect of different control strategies including chemotherapy and vaccination. The model extended the two-host model of Barbour [31] to allow for heterogeneity within human and bovine definitive hosts. It consists of a set of simultaneous equations which model rate of change in prevalence over time.
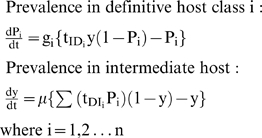
where t_IDi_ and t_DIi_ are composite transmission parameters for intermediate host → ith definitive host and ith definitive → intermediate host transmission respectively, which depend upon duration of infection in the respective hosts, snail and definitive host densities, and force of infection per unit host [28].

The model is parameterised using data of *S. japonicum* epidemiology [21]–[24], including the distribution of endemic prevalences within host classes, and known features of schistosomiasis japonica such as infection duration. Interventions can then be imposed on the system, and the equations solved numerically to predict the consequences for prevalence and incidence.

### Model Parameters and Assumptions

Six endemic scenarios are presented, based on actual baseline epidemiological data collected from villages in an ongoing cluster-randomised intervention trial against *S. japonicum* [24] in the Dongting (Hunan) and Poyang (Jiangxi) lake areas of southern China. Endemic scenarios 1–3 correspond to three of the villages involved in the trial; Yongfu and Mengjiang villages in Hunan Province, and Xindong village in Jiangxi Province. Scenarios 4–6 are based on the same three villages but use a 1.5% water buffalo *S. japonicum* prevalence based on that observed in Samar, the Philippines [32]–[34], so as to simulate transmission under conditions of low *S. japonicum* prevalence in water buffaloes. Table 1 shows the human and water buffalo endemic prevalences and the ratios of numbers of humans to water buffaloes for these scenarios.

**Table 1:** Model parameters and predictions after removal of water buffalo transmission of *S. japonicum* for different endemic scenarios using field epidemiological data from villages in the Dongting (Hunan Province) and Poyang (Jiangxi Province) Lake Areas of Southern China.

Model Parameters	Endemic Scenario					
	1	2	3	4	5	6
Location	Yongfu village, Hunan Province	Mengjiang village, Hunan Province	Xindong village, Jiangxi Province	Yongfu village, Hunan Province	Mengjiang village, Hunan Province	Xindong village, Jiangxi Province
Human *S.j* Prevalence	17.0%	9.5%	14.0%	17.0%	9.5%	14.0%
Buffalo *S.j* Prevalence	18.2%	28.8%	12.2%	a1.5%	a1.5%	a 1.5%
Human: Buffalo Ratio	100∶16.9	100∶11.1	100∶24.5	100∶16.9	100∶11.1	100∶24.5
**Model predictions after removal of water buffalo transmission**						
Human *S.j* Prevalence						
5 years	6.0%	3.2%	4.9%	10.8%	5.8%	7.5%
10 years	1.8%	0.9%	1.5%	6.7%	3.6%	3.9%.
25 years	0.1%	0.0%	0.0%	1.9%	1.1%	0.6%
Human *S.j* Incidence						
5 years	0.1%	0.01%	0.09%	1.9%	0.9%	1.0%
10 years	0.03%	0.0%	0.03%	1.2%	0.6%	0.5%
25 years	0.0%	0.0%	0.0%	0.3%	0.2%	0.1%
Equilibrium *S.j* Prevalence	0.0%	0.0%	0.0%	0.0%	0.0%	0.0%
R_0_	1.453	2.338	1.236	1.134	1.297	1.102
R_1_	0.055	0.02	0.064	0.657	0.790	0.484
B_Tx_	96.2%	99.1%	94.8%	42.1%	39.1%	56.1%

R_0_ = Reproductive Rate (before removal of water buffalo transmission); R_1_ = Reproductive Rate (after removal of water buffalo transmission).

B_Tx_ = Contribution of buffaloes to human *S. japonicum* transmission.

*S.j* = *S. japonicum*.

a Hypothetical value.

The model assumes low *S. japonicum* infection intensity (<100 epg) in water buffaloes for all scenarios along with higher intensities of infection in younger animals (<48 months old) [21]–[24]; a 100∶1 buffalo to human ratio in terms of weight of faecal matter [7], [17]; and 1% *S. japonicum* prevalence in oncomelanid snails across all scenarios. It is also assumed that no cattle are present in the endemic scenarios [21]–[24].

### Model Validation

The mathematical model was used to simulate the cluster-randomised bovine intervention trial [24] for Yongfu village. Actual observed data for Yongfu at baseline and for 3 years of follow-up was used for model validation. The primary aim of the trial is to assess the impact of bovine treatment with praziquantel on human infection rates.

## Results

### Model Predictions

**Figure 2:**
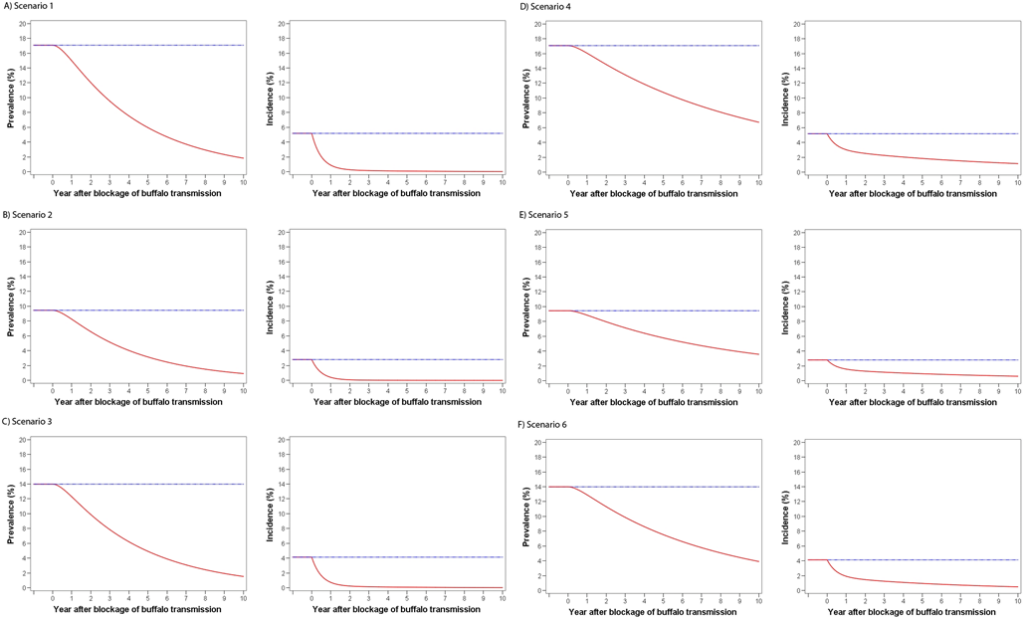
Model predictions of steady-state (continued transmission without intervention) *S. japonicum* transmission (Blue dotted line) and simulation of the removal of *S. japonicum* transmission attributable to water buffaloes (Red solid line). A) Human prevalence and incidence scenario 1 (Yongfu village); B) Human prevalence and incidence scenario 2 (Mengjiang village); C) Human prevalence and incidence scenario 3 (Xindong village); D) Human prevalence and incidence scenario 4 (Yongfu village+hypothetical low *S. japonicum* prevalence in buffaloes; E) Human prevalence and incidence scenario 5 (Mengjiang village+hypothetical low *S. japonicum* prevalence in buffaloes); F) Human prevalence and incidence scenario 6 (Xindong village+hypothetical low *S. japonicum* prevalence in buffaloes.

The predicted steady-state (continued transmission without intervention) *S. japonicum* prevalences and incidences compared to the predicted impact of the removal of schistosome transmission attributable to water buffaloes on human *S. japonicum* prevalence and incidence over time in Yongfu and Mengjiang villages in Hunan Province, and Xindong village in Jiangxi Province for the six different scenarios are shown in Figure 2. Similar results were obtained for all scenarios, with steady-sate *S. japonicum* infection rates remaining constant, while human *S. japonicum* prevalence and incidence were predicted to fall considerably over time, following the removal of *S. japonicum* water buffalo transmission.

Table 1 shows the predicted human infection rates at time points 5, 10 and 25 years post removal of transmission due to water buffaloes, along with the predicted equilibrium prevalence, the parasite reproductive rates pre- and post- removal of water buffalo transmission, and the estimated contribution of water buffaloes to human *S. japonicum* transmission (B_Tx_). All equilibrium prevalences were predicted to be zero and all the parasite reproductive rates fell below 1 following removal of water buffalo transmission. The contribution of water buffaloes to human transmission ranged from 94.8% to 99.1% for scenarios 1–3, and 39.1% to 56.1% for the scenarios (4–6) with the low *S. japonicum* prevalence in water buffaloes.

These scenarios are generalizable to other endemic villages in the lake and marshland areas of China. In these areas, a similar cycle of snail infection and infection/reinfection of humans and water buffaloes occurs [21].

### Model Validation

**Figure 3:**
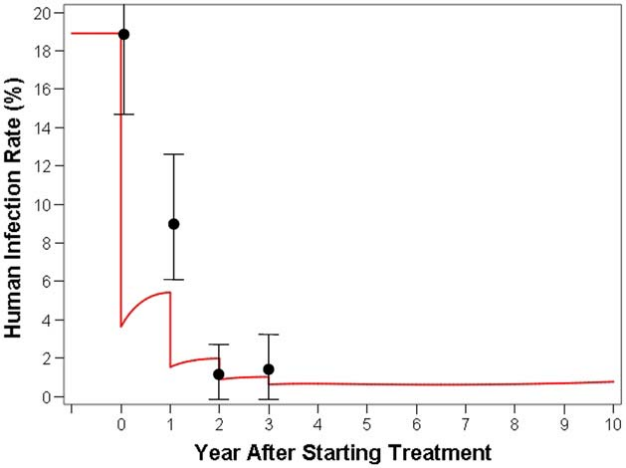
Model Validation—The black dots (with 95% CI) are actual observed data from Yongfu one of the villages involved in the cluster-randomised intervention trial [24] assessing the impact of bovine chemotherapy with praziquantel on human infection rates; the red line shows the simulation of this trial for Yongfu village by the mathematical model.

Validation of the model is shown in Figure 3. Observed results from the cluster-randomised bovine intervention trial for Yongfu village are similar to the predicted results by the model. During the first year of chemotherapy the model predicted a greater reduction in human infection than was actually observed, but this discrepancy is transient.

## Discussion

We used the *S. japonicum* transmission model of Williams *et al* [28] to model the steady-state *S. japonicum* transmission as well as the impact of the removal of *S. japonicum* transmission attributable to water buffaloes on human infection rates across six different endemic scenarios for the Dongting (Hunan) and Poyang (Jiangxi) lakes in southern China. The epidemiological parameters applied to the model used recently collected field data from Hunan and Jiangxi Provinces [24], where most *S. japonicum* transmission occurs in China [7], in order to rationalize model predictions.

The transmission dynamics of schistosomiasis japonica in China can be divided into four main pathways (Figure 1). The bovine component of the pathways is comprised of water buffaloes (*Bubalus bubalis*) and cattle (*Bos taurus*), both of which have similar *S. japonicum* transmission cycles [25]. Water buffaloes, however, spend much more time in water, which is their natural habitat, and so have substantially more exposure to *S. japonicum* than cattle. Laboratory experiments have shown that cattle can be readily infected with *S. japonicum*, but mortality in endemic areas seems to be higher than in water buffaloes [25]–[27], which would explain the greater use of water buffaloes as work animals, particularly for agricultural purposes in China. Such agricultural involvement perpetuates the potential for water buffaloes to contaminate the environment with schistosome eggs compared to cattle [25]–[27].

Blocking either the snail to bovine pathway or the bovine to snail pathway can simulate removal of water buffalo transmission (Figure 1). Here we employed in the model the blocking of the bovine to snail pathway, which prevents miracidia that hatch from eggs excreted by bovines from infecting oncomelanid snail intermediate hosts. This application of the model differs from previous simulations [21], [28], which dealt with specific interventions rather than the complete blockage of a component of the transmission cycle so as to assess and subsequently quantify (B_Tx_) the impact on human transmission. Blockage of the bovine to snail pathway could be achieved by the physical removal of bovines from the environment (a recent pilot initiative employed in China, whereby water buffaloes are being replaced by tractors [35]), bovine praziquantel chemotherapy [10], or deployment of an anti-fecundity (directly) or anti-infection (indirectly) vaccine targeting bovines (particularly water buffaloes) (although vaccines with sufficient efficacy are not yet available [36]).

Because the transmission model is a dynamic process, the effect of the simulation of bovine removal on human infection rates is not immediate and so occurs over time, particularly in relation to prevalence; the effect on incidence is more immediate and pronounced.

In order to account for previous treatment of humans and water buffaloes in the Poyang and Dongting lake areas, low infection intensities were incorporated in all endemic scenarios. This also reduced the transmission pressure applied by water buffaloes, given the 100∶1 buffalo to human stool weight ratio assumed by the model, resulting in the model making more conservative predictions. Furthermore, younger water buffaloes (<48 months) were assigned higher intensities of infection, as there is evidence of a self-cure effect in older animals [37], [38].

The accuracy of the model is supported by the similarity of trial results to model predictions in the praziquantel-based intervention study (1998–2003) around the Poyang Lake in Jiangxi Province [21]. Further model validation is shown in Figure 3 where the observed results of the subsequent cluster- randomised intervention trial [24], assessing the impact of bovine chemotherapy on human infection rates in Yongfu village, are close to model predictions. Results from other intervention studies in relevant settings will also be useful for additional validation.

For endemic scenarios 1–3, the model predicts major reductions in human *S. japonicum* prevalence and incidence following the removal of water buffalo transmission (Table 1; Figure 2). The contribution of water buffaloes to the transmission dynamics of *S. japonicum* was similar, ranging from 94.8%–99.1% (Table 1). This is reflected by the parasite reproductive rates, which were predicted to fall below 1; under 1, the parasite is unable to sustain its population, suggesting that without the contribution of water buffaloes to transmission, *S. japonicum* is unsustainable and cannot remain endemic. This is supported by the equilibrium prevalence (the predicted balance of all prevalences), which was predicted to be zero after the simulation of the removal of schistosome transmission due to water buffaloes.

Endemic scenarios 4–6 used the same parameters as scenarios 1–3, respectively, except for the water buffalo *S. japonicum* prevalence, which was based on that observed in Samar, the Philippines [32]–[34], so as to simulate transmission under conditions of very low (1.5%) *S. japonicum* prevalence. The model also predicted major reductions in human *S. japonicum* prevalence and incidence in scenarios 4–6, albeit slower than predicted for scenarios 1–3, following the removal of water buffalo transmission (Table 1; Figure 2). The predicted contributions of water buffaloes to human transmission were smaller than those of scenarios 1–3, with estimates ranging from 39.1% to 56.1% (Table 1). This is a result of their reduced contribution to the parasite reproductive rate because of low *S. japonicum* prevalence. However, their removal was sufficient to reduce the parasite reproductive rate in the model to below 1, as reflected by the equilibrium prevalences that were predicted to be zero. This indicates that even at low levels of prevalence and infection intensity, water buffaloes are predicted to still be major factors in transmission.

Along with previous epidemiological data, these findings strongly support water buffaloes as an important component of the transmission cycle that affects humans in the lake and marshlands region of China, which appears to differ from the situation prevalent in the Philippines where their contribution is less pronounced. A mathematical model by Riley et al [32], and earlier epidemiological data [33], [34], on which it was formulated, suggest water buffaloes do not play a significant role in the transmission of *S. japonicum* in Samar, the Philippines. This anomaly may be due to parasite genetic variation [32] or differences in cultural, farming and herding practices so that the environmental contamination of schistosome eggs by buffaloes may be less pronounced in the Philippines. Furthermore, the water buffalo (called caribou) in the Philippines is a smaller subspecies (*Bubalus bubalis carabanesis*) that may be less susceptible to schistosome infection. It should be stressed, however, that the procedures for faecal examination of buffaloes in the Philippines are different from those employed in China. In particular, the miracidial hatching test (MHT) [21] used routinely in China, is not used in the Philippines. The MHT involves unmagnified visualization by eye of miracidia hatching from *S. japonicum* eggs in distilled water; 3 hatches (50 g faeces per hatch) are routinely carried out and intensity of infection is then determined by microscopy following a filtration sedimentation procedure similar to the Danish Bilharziasis Laboratory (DBL) technique [39] used in the Samar Province study [33], [34]. Sensitivity analyses have been performed on the DBL-technique [40], but, to date, there has been no direct comparison of its diagnostic performance with that of the MHT. Such a study carried out on buffalo faecal samples from the Philippines would resolve the important issue of the sensitivity of the two tests. If the prevalence for *S. japonicum* in bovines in the Philippines has been underestimated, the conclusions regarding *S. japonicum* transmission dynamics in Samar may need to be revisited. Furthermore, no bovine intervention trials have been performed in the Philippines, and so their true role in *S. japonicum* transmission is not yet known. This is clearly an area for future research being important not only for more fully understanding schistosome transmission dynamics in the Philippines but also for determining appropriate control options there that may involve the treatment and/or vaccination of bovines.

### Final comments

In summary, along with previous epidemiological data, our findings strongly support water buffaloes as an important component of the transmission cycle that affects humans in the lake and marshlands region of China. Our conclusions underscore the rationale for removal, replacement or treatment of water buffaloes, and for the development and deployment of a transmission blocking buffalo vaccine against *S. japonicum* [35], [41] for this setting to achieve the goal of transmission control. The Chinese Government has recently commenced a new integrated national strategy [36] to control resurgent schistosomiasis by: a) reducing bovines and humans as a source of *S. japonicum* infection to snails, b) improving sanitation, c) building lavatories and latrines, and d) providing heavily infected itinerant lake fishermen with fecal containers on their boats so as to reduce the amount of human excreta discharged directly into the lake and marshland areas. This is an important integrated approach that if rapidly and broadly implemented, along with appropriate health education, could achieve China's challenging and ambitious target of reducing the level of human infection in all endemic counties to less than 1% by 2015 [10]. The long term effectiveness and feasibility of this strategy should be further evaluated.
